# Dynamic distribution patterns of ribosomal DNA and chromosomal evolution in *Paphiopedilum*, a lady's slipper orchid

**DOI:** 10.1186/1471-2229-11-126

**Published:** 2011-09-12

**Authors:** Tianying Lan, Victor A Albert

**Affiliations:** 1 Department of Biological Sciences, University at Buffalo, Buffalo, NY 14260, USA

## Abstract

**Background:**

*Paphiopedilum *is a horticulturally and ecologically important genus of ca. 80 species of lady's slipper orchids native to Southeast Asia. These plants have long been of interest regarding their chromosomal evolution, which involves a progressive aneuploid series based on either fission or fusion of centromeres. Chromosome number is positively correlated with genome size, so rearrangement processes must include either insertion or deletion of DNA segments. We have conducted Fluorescence *In Situ *Hybridization (FISH) studies using 5S and 25S ribosomal DNA (rDNA) probes to survey for rearrangements, duplications, and phylogenetically-correlated variation within *Paphiopedilum*. We further studied sequence variation of the non-transcribed spacers of 5S rDNA (5S-NTS) to examine their complex duplication history, including the possibility that concerted evolutionary forces may homogenize diversity.

**Results:**

5S and 25S rDNA loci among *Paphiopedilum *species, representing all key phylogenetic lineages, exhibit a considerable diversity that correlates well with recognized evolutionary groups. 25S rDNA signals range from 2 (representing 1 locus) to 9, the latter representing hemizygosity. 5S loci display extensive structural variation, and show from 2 specific signals to many, both major and minor and highly dispersed. The dispersed signals mainly occur at centromeric and subtelomeric positions, which are hotspots for chromosomal breakpoints. Phylogenetic analysis of cloned 5S rDNA non-transcribed spacer (5S-NTS) sequences showed evidence for both ancient and recent post-speciation duplication events, as well as interlocus and intralocus diversity.

**Conclusions:**

*Paphiopedilum *species display many chromosomal rearrangements - for example, duplications, translocations, and inversions - but only weak concerted evolutionary forces among highly duplicated 5S arrays, which suggests that double-strand break repair processes are dynamic and ongoing. These results make the genus a model system for the study of complex chromosomal evolution in plants.

## Background

*Paphiopedilum*, a genus of approximately 80 species indigenous to tropical and subtropical Southeast Asia, is among the most widely grown and hybridized of all orchids. Species of *Paphiopedilum *are also ecologically important narrow endemics in various mainland and island habitats, which range from montane rainforest to seaside cliffs [1]. Karyological studies of *Paphiopedilum *have revealed considerable chromosomal variation, which ranges from 2n = 26 to 2n = 42, in aneuploid increments suggestive of centric fission [2]. Basic molecular phylogenetic information on the genus is available [3]. Subgenus *Parvisepalum*, which is sister to the rest of the genus, has 2n = 26 metacentric chromosomes, whereas the type subgenus *Paphiopedilum *includes both clades of 2n = 26 species and two distinct lineages of species that bear greater than 26 chromosomes, with the number of telocentrics equal to twice the number of metacentrics that ostensibly split [3]. Haploid genome size is extremely large in these orchids, ranging from 16.1 to 35.1 megabases (Mb)[4]. Chromosome number has been shown to be positively correlated with genome size [4], so rearrangement processes must include either insertion or deletion of DNA segments.

General issues in plant chromosomal evolution include the contribution of rearrangements to genome structure and size. Rearrangement processes involve double-strand break repair, which occurs frequently at hotspots in pericentromeric and telomeric regions [5,6]. Gene duplications may be caused by unequal crossing over, retrotransposition, or genome duplication [7]. Tandem repeats duplication or segmental duplication is one of the possible outcomes of unequal crossing over [7,8]. These phenomena may be investigated empirically through use of Fluorescence *In Situ *Hybridization (FISH) on highly repetitive DNA loci subject to concerted evolution, such as the 18S-5.8S-25S (45S) and 5S ribosomal DNA (rDNA) arrays, which may show duplication or evidence for rearrangement-producing heterologous recombination [9]. Infrageneric comparative rDNA FISH analyses, in which mobility and patterning have been systematically investigated as species-specific karyotype markers, are common in the literature [10-14]. We use such analyses here to document chromosomal dynamics in *Paphiopedilum*. FISH has been applied previously to *Paphiopedilum*, but in a limited manner only, and especially in hybrids [15,16].

Both 45S and 5S rDNAs in plants are characterized by intergenic spacers. 5S rDNA non-transcribed spacer (5S-NTS) sequences have seen some use as phylogenetic markers [17-21]. However, most studies of 5S-NTS to-date have employed direct sequencing of PCR products, and there is evidence that the NTS both within and among arrays can show polymorphism. We have cloned 5S-NTS segments in *Paphiopedilum *in order to study past and ongoing gene duplication events and the possibility of gene conversion both within arrays and among duplicated loci.

We briefly report distribution patterns of rDNA signals from a phylogenetic systematic perspective [22] according to accepted section-level classification. We do not aim to provide complete karyotypic comparisons, nor a full cytotaxonomic treatment; rather, we concern ourselves with demonstrable evidence for dynamic rearrangements during the evolution of *Paphiopedilum*. 5S-NTS sequence data are also compared with a phylogenetic hypothesis in order to ascertain duplication history of paralogs.

## Results

### Distribution patterns of ribosomal DNA by Fluorescence In Situ Hybridization, according to phylogeny and section-level classification

#### Section *Parvisepalum*

**Figure 1:**
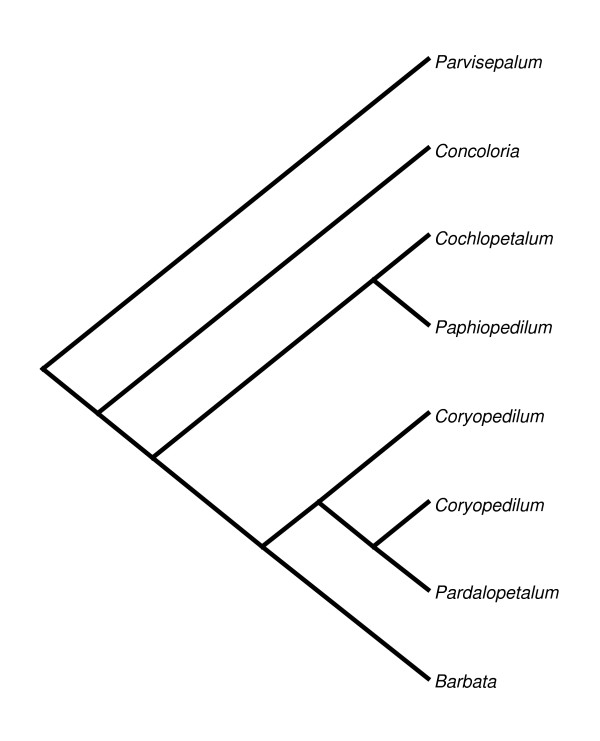
**Section-level phylogenetic tree of genus *Paphiopedilum***. Section-level phylogenetic tree based on rDNA ITS sequences published by Cox [3].

**Figure 2:**
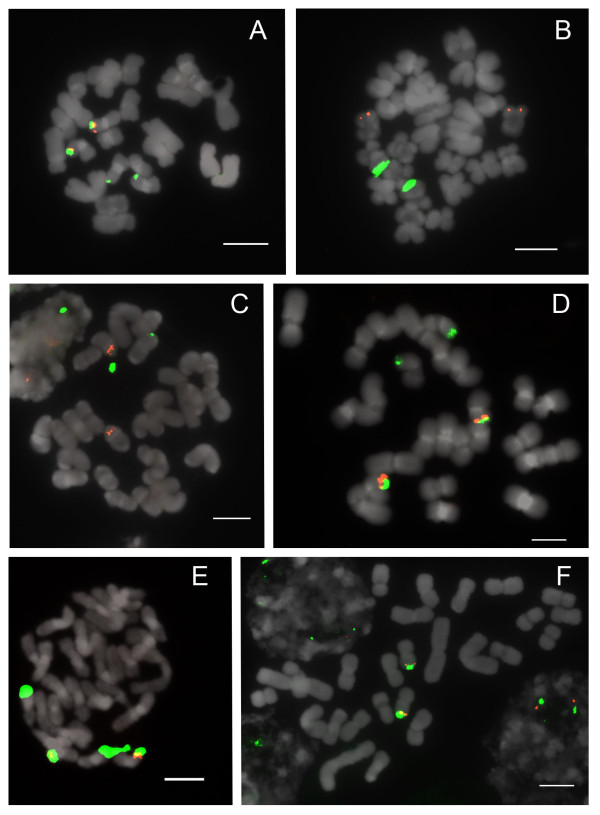
**FISH of 25S and 5S rDNA to metaphase chromosomes of *Paphiopedilum *section *Parvisepalum***. (A) *Paphiopedilum emersonii*, (B) *P. delenatii*, (C) *P. malipoense*, (D) *P. hangianum*, (E) *P. armeniacum*, (F) *P. micranthum*. 25S rDNA (green) and 5S rDNA (red) probes were simultaneously detected in all *Paphiopedilum *species. Chromosomes were counterstained with DAPI. All scale bars = 10 μm.

Section *Parvisepalum *is the sister group of all other *Paphiopedilum *species (Figure 1). Two to four 25S rDNA signals are apparent (Figure 2) among 2n = 26 chromosomes, with two signals most parsimoniously interpretable as the basal condition since this state is shared by the outgroup genera *Mexipedium *and *Phragmipedium *(unpublished data;[23]). With 2 signals being the inferred primitive condition, rearrangement by duplication is observed in *Paphiopedilum armeniacum, P. emersonii *and *P. hangianum*, which have more loci. 5S rDNA patterns are stable, showing 2 subtelomeric signals that are usually closely linked with one pair of 25S signals (Table 1). In *P. delenatii*, translocation of either the 5S or 25S rDNA locus has occurred. This phenomenon is also seen in *P. malipoense*, with its two chromosomes that show hemizygous 25S and 5S rDNA signals, respectively.

**Table 1:** *Paphiopedilum *species studied, diploid chromosome numbers, rDNA FISH patterns, and 5S-NTS sequence polymorphic sites

		Number of rDNA sites				**Positions of rDNA sites**^**b**^		
			
			5S		25S+5S			
							
Taxon	2n	25S	major	**visible sites**^**a**^	Co-localization	5S	25S	5S-NTS Polymorphic sites
*Paphiopedilum*								
Subg. *Parvisepalum*								
Sect. *Parvisepalum*								
*armeniacum*	26	4	2	2	2	st	t	104
*delenatii*	26	2	2	2	0	st	t	178
*emersonii*	26	4	2	2	2	st	t	124
*hangianum*	26	4	2	2	2	st	t	120
*malipoense*	26	2	2	2	1	st	t	94
*micranthum*	26	4	2	2	2	st	t	59
Subg. *Paphiopedilum*								
Sect. *Concoloria*								
*bellatulum*	26	2	2	2	0	i	t	118
*niveum*	26	2	2	2	0	i	t	198
Sect. *Cochlopetalum*								
*liemianum*	32	2	4	22	2	st, i, p, c	t	162
*moquettianum*	34	2	4	20	2	st, i, p, c	t	225
*primulinum*	32	2	4	25	2	st, i, p, c	t	71
*victoria-regina*	34	2	4	24	2	st, i, p, c	t	137
Sect. *Paphiopedilum*								
*druryi*	30	2	4	16	0	st, i, p, c	t	184
*fairrieanum*	26	2	2	14	2	st, i, p, c	t	146
*henryanum*	26	2	2	17	2	st, i, p, c	t	180
*hirsutissimum*	26	2	6	21	2	st, i, p, c	t	182
*tigrinum*	26	2	6	17	2	st, i, p, c	t	141
Sect. *Coryopedilum*								
*adductum*	26	9	4	28	6	st, i, p, c	t, st	180
*gigantifolium*	26	6	6	32	6	st, i, p, c	t	210
*glanduliferum*	26	4	4	26	4	st, i, p, c	t	202
*randsii*	26	4	4	30	4	st, i, p, c	t, st	187
*sanderianum*	26	2	4	16	0	st, i, p, c	t	143
*stonei*	26	2	4	25	2	st, i, p, c	t	114
*supardii*	26	9	4	26	7	st, i, p, c	t	226
Sect. *Pardalopetalum*								
*dianthum*	26	2	4	28	2	st, i, p, c	t	251
*haynaldianum*	26	4	4	8	2	st, i, p, c	t	110
*lowii*	26	6	4	28	4	st, i, p, c	t, st	161
*parishii*	26	4	4	34	4	st, i, p, c	t	189
Sect. *Barbata*								
*acmodontum*	38	2	4	4	0	i	t	169
*curtisii*	36	2	2	2	0	i	t	164
*dayanum*	36	2	4	6	0	i, p	t	169
*hennisianum*	34	2	2	6	0	i	t	153
*purpuratum*	40	2	4	8	0	st, i	t	138
*sangii*	38	2	4	18	0	st, i	t	118
*sukhakulii*	40	2	2	13	0	st, i	t	151
*venustum*	40	2	4	8	2	i	t	109
w*ardii*	42	2	4	4	0	i	t	159

^a ^Minimum numbers of visible 5S rDNA FISH signals, including numbers of both major and visible dispersed sites.

^b ^st, subtelomeric; t, telomeric; i, interstitial; p, pericentromeric; c, centromeric

#### Section *Concoloria*

**Figure 3:**
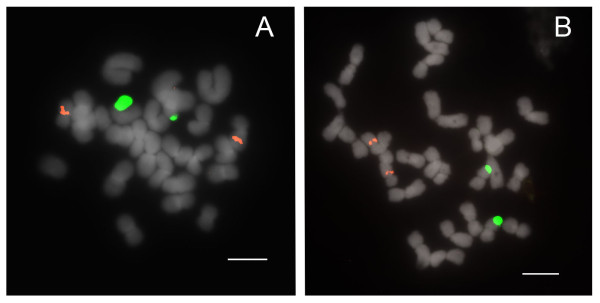
**FISH of 25S and 5S rDNA to metaphase chromosomes of *Paphiopedilum *section *Concoloria***. (A) *Paphiopedilum bellatulum*, (B) *P. niveum*.

Species of section *Concoloria *show two 25S and 5S signals (Table 1), each on separate chromosomes (2n = 26 total), similarly to *Paphiopedilum delenatii *of section *Parvisepalum*, except in that the 5S signals are interstitially instead of subtelomerically placed (Figure 3).

#### Section *Cochlopetalum*

**Figure 4:**
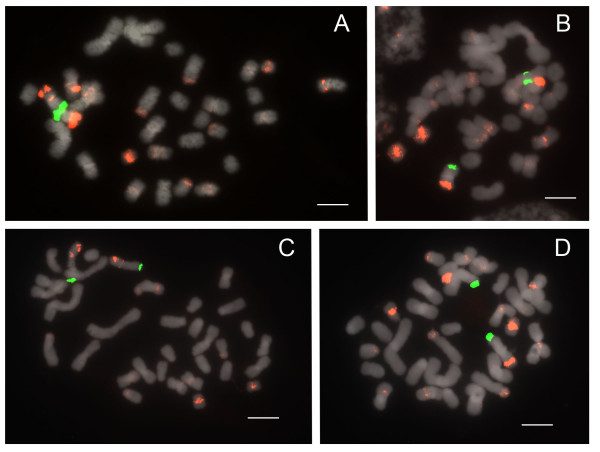
**FISH of 25S and 5S rDNA to metaphase chromosomes of *Paphiopedilum *section *Cochlopetalum***. (A) *Paphiopedilum primulinum*, (B) *P. moquettianum*, (C) *P. liemianum*, (D) *P. victoria-regina*.

Section *Cochlopetalum *displays an aneuploid number of chromosomes, the telocentrics of which have been suggested to descend via centric fission from 25 diploid metacentrics [2]. According to phylogenetic relationships known at present (Figure 1), and the centric fission hypothesis, sections *Cochlopetalum *and *Barbata *(with telocentrics descended from 26 diploid metacentrics) have evolved aneuploid increase independently. All four species studied here have two telomeric 25S rDNA signals, and 4 major 5S rDNA signals (Figure 4; Table 1). All 4 species have multiple dispersed 5S signals, rather unlike species of sections *Parviflora *and *Concoloria*, and these, like the major loci, are mostly subtelomeric, pericentromeric and centromeric in position. The 2 species with 2n = 32 chromosomes, *Paphiopedilum liemianum *(Figure 4C) and *P. primulinum *(Figure 4A), both have two 5S bands localized on the same chromosomes as the 25S signals, whereas only a single 5S band is seen on the same chromosome in the 2n = 34 species *P. moquettianum *(Figure 4B) and *P. victoria-regina *(Figure 4D).

#### Section *Paphiopedilum*

**Figure 5:**
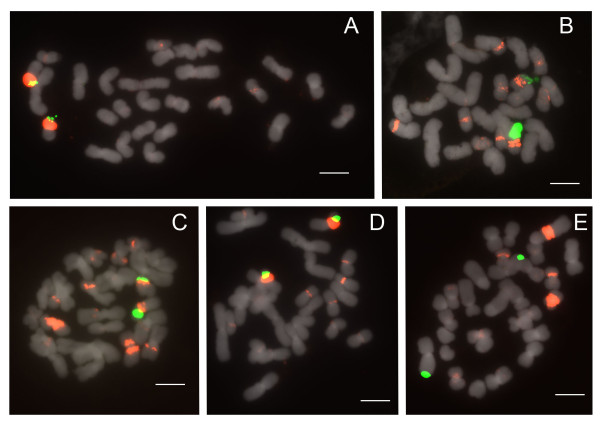
**FISH of 25S and 5S rDNA to metaphase chromosomes of *Paphiopedilum *section *Paphiopedilum***. (A) *Paphiopedilum fairrieanum*, (B) *P. hirsutissimum*, (C) *P. tigrinum*, (D) *P. henryanum*, (E) *P. druryi*.

All 5 species of section *Paphiopedilum *studied show two 25S signals in the telomeric region (Figure 5; Table 1). All species, which are 2n = 26 except for *P. druryi *(Figure 5E) at 2n = 30, show at least 2 specific 5S rDNA bands, as many as 6, and numerous dispersed signals in the pericentromeric and centromeric regions. In all but *P. druryi *the major signals are closely linked with the 25S arrays. In *P. druryi*, 4 of the major signals appear to be located on different arms and on morphologically different chromosomes that may only be partly homologous (this condition was observed in at least 4 cells).

#### Sections *Coryopedilum *and *Pardalopetalum*

**Figure 6:**
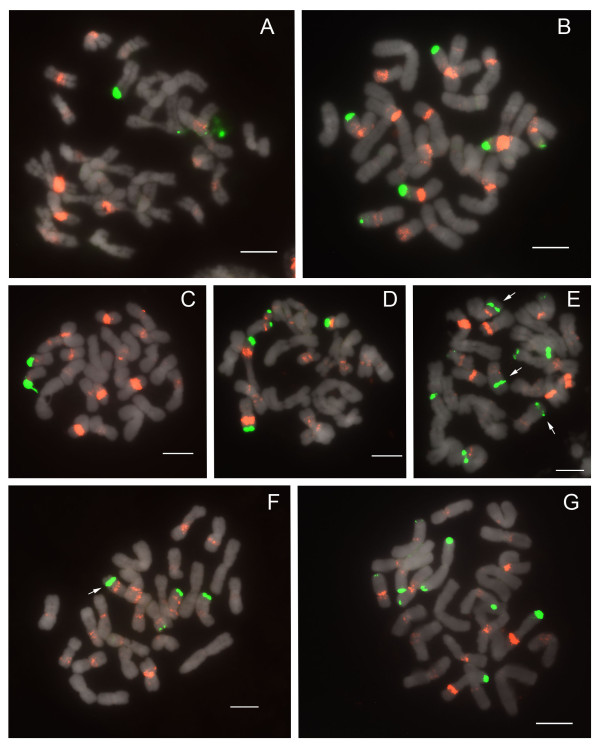
**FISH of 25S and 5S rDNA to metaphase chromosomes of *Paphiopedilum *section *Coryopedilum***. (A) *Paphiopedilum sanderianum*, (B) *P. gigantifolium*, (C) *P. stonei*, (D) *P. glanduliferum*, (E) *P. adductum*, (F) *P. randsii*, (G) *P. supardii*. Arrows indicate subtelomeric 25S rDNA signals.

**Figure 7:**
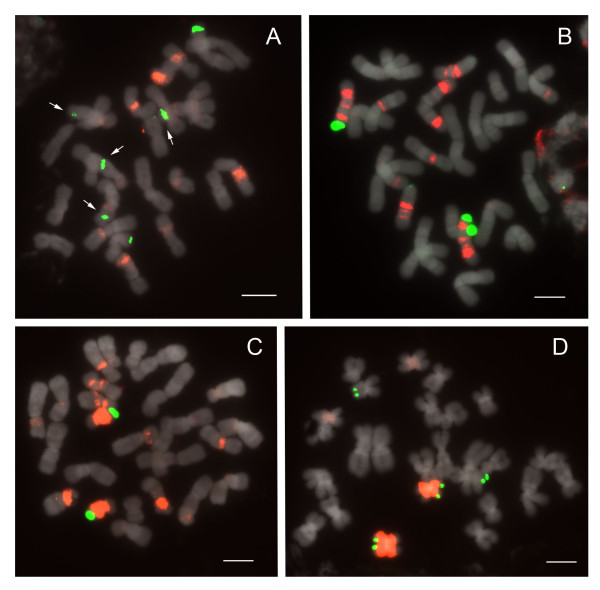
**FISH of 25S and 5S rDNA to metaphase chromosomes of *Paphiopedilum *section *Pardalopetalum***. (A) *Paphiopedilum lowii*, (B) *P. parishii*, (C) *P. dianthum*, (D) *P. haynaldianum*. Arrows indicate subtelomeric 25S rDNA signals.

In current phylogenetic results, section *Pardalopetalum *is derived within section *Coryopedilum *(Figure 1); as such, they will be discussed together here. Together, the *Coryopedilum/Pardalopetalum *clade, all species having 2n = 26, is the most dynamic in *Paphiopedilum *regarding chromosomal rearrangements (Figure 6, 7; Table 1). 25S signals vary from 2 to 9, the latter showing hemizygosity. Signals in all species except *Paphiopedilum lowii *(Figure 7A), *P. adductum *(Figure 6E) and *P. randsii *(Figure 6F) are telomeric. 1-4 subtelomeric 25S signals were observed in *P. lowii, P. adductum *and *P. randsii*. In *P. supardii *(Figure 6G), one hemizygous chromosome has telomeric 25S signals on each arm. *P. adductum *also shows 25S hemizygosity, and both this species and *P. supardii *show the maximum number of signals. Species of the *Coryopedilum/Pardalopetalum *group show at least 4 major 5S rDNA signals (up to 8 in *P. parishii *(Figure 7B)) and multiple dispersed repeats in pericentromeric and centromeric regions. In the *Pardalopetalum *group, all species show at least 2 strong (up to 5) 5S bands located on one chromosome. Close linkage with 25S occurs throughout the group, other than in *P. sanderianum *(Figure 6A), either with major or minor 5S bands, and appearing in different placements along chromosome arms.

#### Section *Barbata*

**Figure 8:**
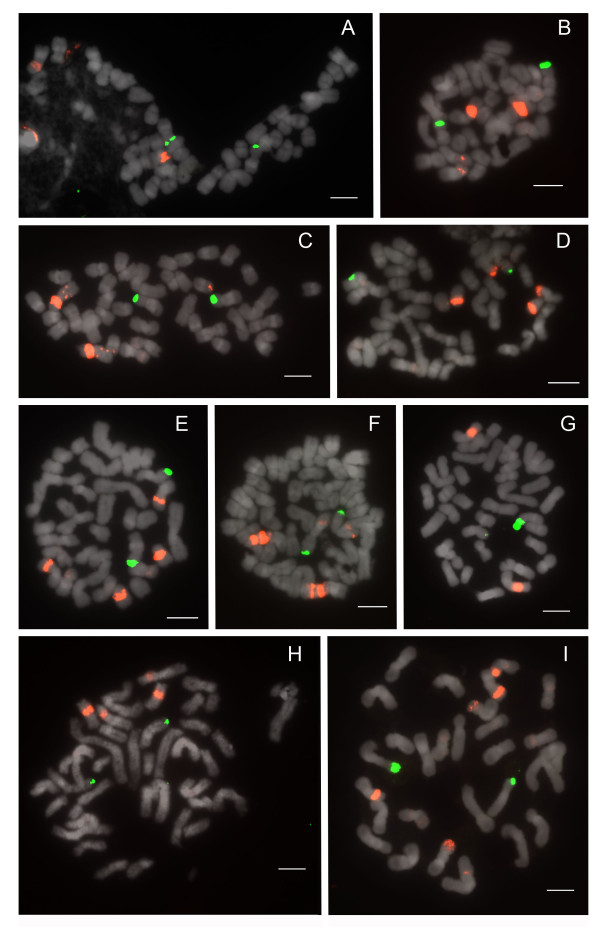
**FISH distribution pattern of 25S and 5S rDNA on metaphase chromosomes of *Paphiopedilum *section *Barbata***. (A) *Paphiopedilum wardii*, (B) *P. hennisianum*, (C) *P. sukhakulii*, (D) *P. purpuratum*, (E) *P. dayanum*, (F) *P. venustum*, (G) *P. curtisii*, (H) *P. acmodontum*, (I) *P. sangii*.

Species of section *Barbata*, which have 2n = 28-42 and the largest genome sizes, show constancy in 25S rDNA distribution, with 2 telomeric signals (Figure 8; Table 1). Major 5S signals number 2-4, and extremely few dispersed repeats were observed. Most 5S loci are not centromeric, whereas telomeric, subtelomeric, pericentromeric, and interstitial placements are observed. Only *Paphiopedilum curtisii *(Figure 8G) and *P. hennisianum*(Figure 8B) have two major 5S signals, and the first species shows no dispersed repeats. *P. sukhakulii *(Figure 8C), *P. venustum *(Figure 8F) and *P. wardii *(Figure 8A) show linked 5S signals. Only in *P. venustum *is close linkage of 25S and 5S observed, and then only involving a minor 5S band. Because *Barbata *is the most derived section in the genus (Figure 1), either its species have lost 25S and 5S rDNA loci, since *Cochlopetalum, Paphiopedilum, Coryopedilum*, and *Pardalopetalum *usually have more, or the species of the latter sections have increased the number of rDNA loci independently given the low number in sections *Parvisepalum *and *Concoloria*.

### Diversity of 5S ribosomal DNA non-transcribed spacer sequences

**Figure 9:**
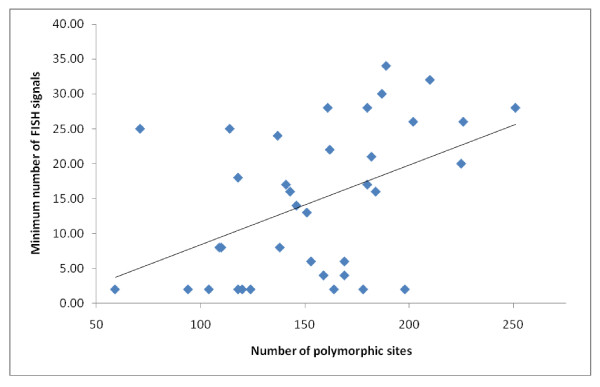
**The relationship between polymorphism of 5S-NTS sequences and numbers of observed 5S rDNA signals**. Line indicates trend derived from linear regression analysis based on 5S-NTS within-species polymorphic sites and minimum numbers of visible 5S signals (data from Table 1.). *P *< 0.01, R^2 = 0.21.

We investigated duplication history correlated with the dynamic rearrangements observed in 5S rDNA loci. In order to survey sequence variation in 5S-NTS, random clones, 7 (*Paphiopedilum niveum*) or 8 (all others) per species, were sequenced (Additional file 1). Only a few clones were identical to each other (2 sequences from *P. acmodontum*, 2 from *P. henryanum*, 2 from *P. hirsutissimum*, 2 from *P. stonei*, 4 from *P. dayanum*, 4 from *P. malipoense*, and one sequence each of *P. stonei *and *P. supardii*). Sequences of 5S-NTS ranged from 283 bp (*P. micranthum *1) to 455 bp (*P. bellatulum *5). Given extensive sequence divergence of 5S-NTS and our desire not to manually adjust alignment [24], an objective alignment was accomplished using MAFFT and default settings. Numbers of polymorphic loci within species, and phylogenetic relationships, were assessed in order to estimate the strength of gene conversion and the extent of paralogy, respectively. Numbers of polymorphic sites within species positively correlated with minimum numbers of visible 5S signals (*P *< 0.01, R^2 = 0.21; Figure 9), suggesting that interlocus gene conversion is relatively weak. A phylogenetic tree outgroup-rooted using *Phragmipedium besseae *showed 2 major groups of sequences: section *Parvisepalum *versus the remainder of the genus. The single tree of maximum likelihood is shown (as a phylogram, Additional file 2), as is the majority-rule consensus tree based on 100 bootstrap replicates (Additional file 3). Some large species-specific clades were observed, as well as some section-specific clades. Overall, however, the phylogenetic tree was poorly representative of phylogenetic relationships due to extensive duplication of 5S loci.

## Discussion

### Variation in numbers and chromosomal locations of rDNA

Variation in numbers and distribution patterns of rDNA loci among related species is commonly observed in many different plant genera, including Brassicaceae [10], Cyperaceae [11], Asteraceae [25,26], Leguminosae [27], *Pinus*[28], and Rosaceae [14]. Plants typically show some degree of conservatism of rDNA repeat duplication, such that when multiple loci do appear, species are commonly polyploid relatives of diploids. There is no evidence at all, however, for polyploidy in *Paphiopedilum*, where the only chromosome number differences are aneuploid, in a series reflective of centric fission or fusion.

In general, FISH patterns of 25S rDNA loci are reported to be more polymorphic than those of the 5S rDNA [12-14,26,28-32]. Conversely, in all sections of *Paphiopedilum*, except for *Parvisepalum *and *Concoloria*, 5S rDNA sites showed much more variability both in number and physical location than did 25S rDNA sites.

The most parsimonious ancestral number of 25S rDNA sites in *Paphiopedilum *is two, based on outgroup comparison to the genera *Mexipedium *and *Phragmipedium *(unpublished results;[3,22]). Duplication of 25S rDNA sites was observed only in three of the seven sections of *Paphiopedilum*: *Parvisepalum *(2n = 26), *Coryopedilum *(2n = 26) and *Pardalopetalum *(2n = 26) (Table 1). The physical positions of 25S rDNA loci are relatively conservative. In most *Paphiopedilum *species we analyzed, 25S rDNA signals are located in terminal chromosome positions. Variation was only observed in three species, *Paphiopedilum adductum, P. randsii *and *P. lowii*, which showed 1-4 subtelomeric 25S rDNA signals (Figures 6E, F and 7A, respectively). The ancestral number of 5S rDNA sites, again by outgroup comparison, is 2 (unpublished results from *Mexipedium *and *Phragmipedium*), and is only observed in sections *Parvisepalum *and *Concoloria*. Massive duplication and amplification of 5S rDNA loci, leading to large-scale polymorphism of numbers, sizes and physical positions of signals, was found prevalent in the remaining five sections. The numbers and distribution of rDNA loci vary widely among plants; however, usually less than one-third of chromosomes display either 45S rDNA or 5S rDNA [13]. It is therefore noteworthy that in some lineages of *Paphiopedilum*, up to 24 of the 26 chromosomes bear at least one rDNA locus, and a single chromosome can bear up to five major 5S rDNA loci.

Apparently, there is no strong correlation between the increase in the number of rDNA sites and the increase in the number of chromosomes or genome size. A similar situation has also been described in many other diploid species, e.g. the diploid lineage of Brassicaceae [10], Cyperaceae [11,12], *Iris*[13], and Rosaceae [14]. The massive duplication of rDNA loci in *Paphiopedilum *sections *Cochlopetalum, Paphiopedilum, Coryopedilum *and *Pardalopetalum *could partly contribute to the increase of genome size. Perhaps paradoxically, species with the smallest (*P. exul; *section *Paphiopedilum*) and largest (*P. dianthum*; section *Pardalopetalum*) haploid genome sizes are both members of groups that show considerable 25S and 5S locus duplication in our FISH experiments. These two species differ more than two-fold in genome size, 16.1 to 35.1 Mb, respectively [33]. If we assume that the number of distinct genes among *Paphiopedilum *species is roughly constant, this would suggest that genome size increase is primarily due to repetitive element amplification, but that since rDNA duplication is associated with both smaller *and *larger genomes in the genus, size differences may be more logically traceable to other repetitive DNAs, such as mobile elements. However, a possible tendency for elimination of rDNA loci was found in section *Barbata*, which has the greatest average genome sizes and chromosome numbers [4]. The number of 25S rDNA loci in *Barbata *remains two through all the species we studied, while the distribution pattern of 5S rDNA is less dispersed than its sister group, *Coryopedilum *plus *Pardalopetalum*. Due to the derived phylogenetic position of section *Barbata *(Figure 1), it is most parsimonious to conclude that unique chromosomal conditions seen in the group would be similarly derived (autapomorphic). As such, centric fission in *Barbata *appears to be associated with loss of rDNA loci, while in other systems, centric fission has led to rDNA gains [34]. Elimination of rDNA loci during chromosomal evolution has been documented in, e.g., Brassicaceae and Rosaceae [10,14]. The mechanism that accounts for such loss of rDNA loci, however, remains unclear. A presumed evolutionary loss of abundant terminal nucleolar organizing regions (NOR) in *Arabidopsis *has been hypothesized to be the consequence of an ancient fusion event [35]. In the case of section *Barbata*, additional traceable chromosome markers are needed to provide further evidence that chromosomal rearrangements are related to rDNA loss.

### A combination of different mechanisms causes high mobility of rDNA

Different mechanisms have been postulated to account for the mobility and polymorphism of numbers, sizes and positions of rDNA sites, such as transposon-mediated transpositional events [36-38], and chromosome rearrangements (translocation, inversion, duplication, deletion) caused by homologous or non-homologous unequal crossing-over and gene conversion [9,28,30,36]. These processes could act alone or in combination, and they do not necessarily imply changes in overall chromosome morphology [31,34].

The great degree of 5S repeat dispersion seen in sections *Cochlopetalum, Paphiopedilum, Coryopedilum *and *Pardalopetalum *has, to our knowledge, only been observed in the monocots *Alstroemeria, Tulipa*, and *Iris*[13,39,40]. The original seeding of rDNA repeats to ectopic locations in the genome could be the result of transposable element activity or perhaps incorporation of array segments into breakpoints as part of non-homologous end joining during DNA repair. Indeed, some of the signals we observed may be pseudogenes transported within the genomes by retroelements, therefore leading to the false interpretation that we are visualizing entire and active rDNA arrays. Both subtelomeric and pericentromeric regions are well known as hot spots of breakpoints and are also enriched for TEs [5,6]. Considering the abundant minor loci we observed in these regions, a contribution of transpositions to the dispersed distribution pattern is tenable, and TEs containing 5S rDNA-derived sequences have in fact been observed in many plants [41] and animals [42]. It is nonetheless possible that due to the similarity of rDNA arrays, chromosomal rearrangement could be induced via heterologous recombination, and in turn, rearrangement could generate repeated sequences through unequal crossovers. After generation of a novel locus, *in situ *amplification cycles via rearrangement could lead to the origin of FISH-detectable loci. Furthermore, hemizygous 5S rDNA sites have been widely observed in many *Paphiopedilum *species. A double-strand break occurring in a hemizygous region would increase the probability of causing other rearrangements, owing to the absence of a homologous template for its repair [5]. The lack of dispersed repeats in the basalmost section *Parvisepalum *may reflect either a lack of seeding events or slow amplification processes that do not yield hybridization-visible arrays. However, in the case of 5S rDNA, there is in fact strong evidence for NTS sequence diversity, which could either be accounted for by the presence or small loci below the FISH detection limit or perhaps by considerable within-array diversity. One future experimental approach to determine whether considerable intra-array diversity indeed exists would be to perform FISH using 5S-NTS-specific probes.

Diversification of 25S rDNA distribution patterns is also observed in *Paphiopedilum*, but the numbers of loci and degree of dispersion is much lower than for 5S rDNA. Therefore, 5S rDNA might be more frequently seeded by TEs via transpositional events, or, amplification or maintenance of 5S rDNA loci via rearrangement could be more effective and tolerated during the chromosome evolution process. The different evolutionary tendencies between 25S and 5S rDNA might be caused by their function and sequence divergence or localization in distinct nuclear compartments [43].

### 5S-NTS sequences highlight interlocus and intralocus diversity and weak concerted evolutionary forces

Previous studies of other angiosperm species have suggested that intralocus 5S rDNA diversity occurs. Within-array 5S rDNA diversity appears very likely in *Paphiopedilum *as well, since many species (e.g., all *Parvisepalum *and *Concoloria*) have only one observable 5S locus. For example, 6 species of section *Parvisepalum *are represented in our phylogenetic analysis by 6-8 distinct sequence variants. These 5S-NTS variants can be concluded to occur within at least partial arrays, pseudogenized or not, since the amplified pieces include sections of 5S rDNA at their 5' and 3' ends. Recent within-species duplication events may be indicated by single-species clades of 5S-NTS sequences, such as *P. dayanum, P. lowii, P. sangii*, but these could just as well indicate within-array variation, as single-species clades of *Parvisepalum *(e.g., *P. malipoense*) and *Concoloria *(*P. bellatulum*) most likely do. In many cases, it can be readily seen that duplication of 5S loci has occurred prior to speciation, for example, within *Coryopedilum *(a large group of sequences representing *P. sanderianum, P. stonei*, and *P. supardii*; similarly also within a group of *P. adductum *and *P. randsii *sequences). In some cases, ancient duplications must be much older than the major phylogenetic groups of *Paphiopedilum*, since, for example, *P. delenatii *shares sequence variants similar to other *Parvisepalum *species yet has at least one other variant that is more similar to sequences from all other sections. We investigated the possibility of contamination regarding this finding, but discovered similar repeats across 8 distinct *P. delenatii *accessions (results not shown). Another explanation for multispecies clades, e.g., within well-defined groups such as *Parvisepalum *could be ancient hybridization.

We observed that increasing within-species 5S NTS sequence diversity correlates with increasing minimum numbers of visible 5S rDNA loci in *Paphiopedilum *(Figure 9); therefore we infer that interlocus concerted evolution is weak within the genus. Our conclusion concurs with previous findings in many plant genera, such as *Gossypium*[17], *Triticum*[18], *Chenopodium*[19], *Nicotiana *[20] and *Pinus*[27]. So far, to our knowledge, noticeable interlocus concerted evolution of 5S rDNA arrays has not been demonstrated in plants.

The best supported hypothesis to explain weak homogenization forces on 5S rDNA arrays is that the chromosomal location of rDNA arrays has a substantial impact on interlocus concerted evolution [17,20,44-47]. Arrays located in subtelomeric regions are thought to undergo stronger interlocus homogenization forces than ones located in proximal regions. Potential evidence was observed in section *Barbata*, in which all of six species studied possess two 5S loci. These six species can be categorized into two groups according to the locations of 5S loci. One group harboring proximal 5S loci includes *P. wardii, P. dayanum, P. venustum *and *P. acmodontum*, while the other group harboring subtelomeric 5S loci includes *P. purpuratum *and *P. sangii *(Figure 8; Table 1). Considering that all six species are closely related and possess the same number of loci, it can be logically assumed that the difference in sequence polymorphism between the two groups is caused by the different locations of the 5S loci. The fact that the average number of polymorphic sites in the proximal-loci group (151.5) is 18% more than that in the subtelomeric-loci group (128), indicates that proximally located loci seem less homogenized than the subtelomerically located loci.

Additionally, we found that not only interlocus but also intralocus concerted evolution is also influenced by chromosomal localization. In section *Concoloria*, two closely related species, *P. bellatulum *and *P. niveum*, both have one 5S locus, but with different localizations. The difference in sequence polymorphism between the two species may be caused by the different locations of the 5S loci. *P. niveum*, which has a pericentromeric locus, showed 1.68 - fold more polymorphic sites than *P. bellatulum*, which has a subtelomeric locus (Table 1).

It is well-known that meiotic homologous recombination has been largely suppressed in pericentromeric and centromeric regions. Unequal crossovers between sister chromatids and gene conversion documented in the centromeres of many organisms have been postulated as the major homogenization force for tandem repeats located in these areas [48-52]. A plausible explanation for this has been proposed previously: if unequal crossover events between rDNAs of two chromosomes occurred in the proximal region to centromeres, this may result in the exchange of not only a fraction of the rDNA but also the centromeres themselves. Such an event is more likely to have significantly greater negative consequences to the organism than if the event occurred in the subtelomeric region, which then might result in exchange of telomeres [17,47]; loss of centromeres would prohibit cell division, whereas loss of telomeres might not restrict mitosis or meiosis. As such, centromerically-located rDNA arrays are expected to show weaker homogenization forces, since fewer individuals with unequal crossovers in this region are expected to survive. In contrast, the subtelomeric region is characterized by a higher rate of interchromosomal exchange [5], thus stronger concerted evolutionary forces could be expected in this region.

All species of section *Parvisepalum*, as with *P. bellatulum*, have subtelomeric 5S loci, some of which are closely linked with 25S loci. If 5S localization correlates significantly with homogenization, as with 25S, which is always telomeric-subtelomerically located, we should expect subtelomeric 5S repeats to show decreased sequence diversity due to stronger concerted evolutionary forces. However, this is not the case, since variation in the number of polymorphic sites is not significantly different by section (with or without *Pardalopetalum *included in *Coryopedilum*; single factor ANOVA *P *= 0.06 and 0.1, respectively). We therefore infer that localization of the 5S rDNA arrays only partially contributes to the weak concerted evolution observed in *Paphiopedilum*.

There are several other hypothesized mechanisms that could lead to the weak concerted evolutionary force on 5S rDNA arrays. For example, ongoing chromosomal rearrangement such as insertion, deletion, or transposition could occur within arrays too frequently for interlocus concerted evolution to be effective. Another possibility is that concerted evolutionary processes homogenize 5S rDNA arrays at rates lower than the rate of speciation, thus novel mutations cannot be fixed or removed and high levels of intralocus polymorphism are expected within arrays [17]. Additionally, the base composition and secondary structure of rDNA sequences may also affect the rate of concerted evolution [53]. It is unknown whether weak concerted evolutionary forces are shared by other *Paphiopedilum *tandem repeats, or if this is characteristic of 5S rDNA arrays only. This issue can be elucidated by further studies on other tandem repeats, such as 25S rDNA arrays.

## Conclusions

*Paphiopedilum *species display many chromosomal rearrangements - for example, duplications, translocations, and inversions - but only weak concerted evolutionary forces among highly duplicated 5S arrays, which suggests that double-strand break repair processes are dynamic and ongoing. These results make the genus a model system for the study of complex chromosomal evolution in plants.

## Methods

### Plant materials

Thirty-seven species of the *Paphiopedilum *genus covering all seven sections were analyzed in this study. Information on the species and sections is provided in Table 1. Actively growing roots were used for chromosome preparation, while leaves were used for genomic DNA extraction.

### Chromosome preparation

Root tips were pre-treated with 0.004 M 8-hydroxyquinoline for 4-6 h at 10°C, and fixed in freshly prepared fixative (3:1 ethanol: acetic acid) for 48 h at 10°C. The fixed root tips were then rinsed thoroughly with tap water and macerated in an enzyme mixture containing 2% cellulase (Onozuka R-10, Rpi) and 1% pectolyase (*Aspergillus japonicus *Y-23, MP) at 37°C for 30 min. After re-fixation in fixative for 15 min, the meristematic cells were squashed in a drop of 45% acetic acid under a coverslip (22 × 22 mm) on a microscope slide. Slides were then dipped into liquid nitrogen and air dried after the coverslips were carefully removed by a blade.

### Probe labelling and Fluorescence in situ hybridization (FISH)

25S rDNA, a 2.3-kb *Cla*I subclones of the 25S rDNA coding region of *Arabidopsis thaliana *[54] and 5S rDNA (pTa794) [55]were used as probes. 25S rDNA was labelled with biotin-16-dUTP (Roche) and 5S rDNA was labelled with digoxigenin-11-dUTP (Roche), all by nick translation method using the kit from Roche. The hybridization buffer consisted of 50% deionized formamide, 2 × SSC, 50 mM sodium phosphate (pH 7.0), 10% dextran sulfate and sheared salmon sperm DNA (Invitrogen) in 100 × excess of labeled probes. The 25S and 5S rDNA probes were mixed to a final concentration of about 2 ng/μl and then denatured at 94°C for 10 min before being used. Slides with metaphase spreads were treated with 70% deionized formamide in 2 × SSC at 70°C for 2 min. Denatured probes in hybridization buffer were then applied to the slides, which were incubated at 37°C for 10 h in a humid chamber. Post-hybridization washes and immunodetection were carried out in an automated in situ hybridization instrument, the InsituPro VSi (Intavis Bionanalytical Instruments). The slides were washed in 2 × SSC at room temperature for 5 min and twice in 2 ×SSC at 50°C for 10 min. Fluorescence signal was detected using anti-Digoxigenin-Rhodamine conjugate (Roche) and streptavidin-fluorescein conjugate (Invitrogen). The preparations were mounted and counterstained in Vectashield containing 1.5 μg/ml DAPI (4', 6-diamidino-2-phenylindole) (Vector Laboratories). Images were taken by a Zeiss AxioCam MRm black-and-white CCD camera on a Zeiss Imager. Z1 fluorescence microscope and then processed uniformly using Zeiss AxioVision software. FISH signals were false-colored, and DAPI fluorescence was left in gray-tone.

### PCR amplification, cloning and sequencing

Total genomic DNA was extracted from fresh leaves using Qiagen DNeasy Plant Mini kit. The 5S-NTS region was amplified by PCR using the universal degenerate primers: 5'-TGGGAAGTCCTYGTGTTGCA-3' and 5'-KTMGYGCTGGTATGATCGCA-3'[56]. Touchdown amplification was performed as follows: an initial step at 94°C for 5 min, followed by 10 cycles of 94°C for 1 min, annealing for 1 min (start at 60°C, and decreased by 1°C per cycle), and 72°C for 1 min, then 35 cycles of 94°C for 1 min, 50°C for 1 min, and 72°C for 1 min, the final step at 72°C was extended to 10 min. After gel purification using QIAquick Gel Extraction Kit (Qiagen), PCR products were ligated into pDrive Cloning vector and transformed into QIAGEN EZ competent cells (Qiagen PCR Cloning kit). Recombinant clones were screened by colony direct PCR method and were sequenced 7-8 clones per each species using T7 (5'-TAATACGACTCACTATAGGG-3') primer.

### Data analysis

Sequences were aligned using the MAFFT (Multiple Alignment using Fast Fourier Transform) web server at the European Bioinformatics Institute [57]. Default parameters were used: gap opening penalty = 1.53, gap extension penalty = 0.123, tree rebuilding number = 1, maxiterate = 0, and perform FFTS = localpair. The sequence alignment is available as a supplementary FASTA file (Additional file 4).

Within-species 5S-NTS polymorphism was estimated, based on the aforementioned multiple alignment, using DnaSP version 5.10.01 [58]. The relationship between numbers of polymorphic sites and minimum numbers of visible 5S rDNA signals was investigated using linear regression analysis (in Microsoft Excel).

Phylogenetic reconstruction was performed using maximum likelihood optimization available through the RaxML BlackBox web server [59] running RaxML version 7.2.8 [60]. Default settings were used. The 8 *Phragmipedium besseae *sequences were indicated as the outgroup. RaxML was called using the following commands: raxml -# 100 -n pasted -o bess1, bess2, bess3, bess4, bess5, bess6, bess7, bess8 -f a -m GTRGAMMA -x 564547904 -p 564547904 -s 0VaDTW. All search information, as was output on the web site, is included in Additional file 5.

## Authors' contributions

TL and VAA conceived of the study, TL performed all experiments, TL and VAA analyzed data, and both TL and VAA prepared the manuscript. All auhors read and approved the final manuscript.

## Supplementary Material

Additional file 1 GenBank data deposition information of 5S-NTS sequences Click here for file

Additional file 2 5S-NTS sequences: the single tree of maximum likelihood Click here for file

Additional file 3 5S-NTS sequences: the majority-rule consensus tree based on 100 bootstrap replications Click here for file

Additional file 4 The 5S-NTS sequence alignment using MAFFT, provided in FASTA format Click here for file

Additional file 5 Report from RAxML phylogenetic analysis of 5S-NTS sequences Click here for file
