# Supplementary material for: Dynamic distribution patterns of ribosomal DNA and chromosomal evolution in Paphiopedilum, a lady's slipper orchid
Source: BMC Plant Biol. 2011 Sep 12;11:126. doi: 10.1186/1471-2229-11-126 (PMC3184063; doi:10.1186/1471-2229-11-126)
Supplement: Additional file 1 — GenBank data deposition information of 5S-NTS sequences [file 1471-2229-11-126-S1.DOCX]

| Taxon | Abbreviation | GenBank accession number |
| --- | --- | --- |
| *Paphiopedilum* |  |  |
| Subg. *Parvisepalum* |  |  |
| Sect. *Parvisepalum* |  |  |
| *armeniacum* | arm | JN117307 – JN117314 |
| *delenatii* | del | JN117347 – JN117354 |
| *emersonii* | emer | JN117371 – JN117378 |
| *hangianum* | hang | JN117403 – JN117410 |
| *malipoense* | mal | JN117459 – JN117466 |
| *micranthum* | micr | JN117467 – JN117474 |
| Subg. *Paphiopedilum* |  |  |
| Sect. *Concoloria* |  |  |
| *bellatulum* | bell | JN117315 – JN117322 |
| *niveum* | niv | JN117483 – JN117489 |
| Sect. *Cochlopetalum* |  |  |
| *liemianum* | liem | JN117443 – JN117450 |
| *moquettianum* | moq | JN117475 – JN117482 |
| *primulinum* | prim | JN117498 – JN117505 |
| *victoria-regina* | vict | JN117578 – JN117585 |
| Sect. *Paphiopedilum* |  |  |
| *druryi* | dru | JN117363 – JN117370 |
| *fairrieanum* | fair | JN117379 – JN117386 |
| *henryanum* | henry | JN117427 – JN117434 |
| *hirsutissimum* | hirs | JN117435 – JN117442 |
| *tigrinum* | tigr | JN117562 – JN117569 |
| Sect. *Coryopedilum* |  |  |
| *adductum* | add | JN117299 – JN117306 |
| *gigantifolium* | gig | JN117387 – JN117394 |
| *glanduliferum* | glan | JN117395 – JN117402 |
| *randsii* | rand | JN117514 – JN117521 |
| *sanderianum* | sand | JN117522 – JN117529 |
| *stonei* | ston | JN117538 – JN117545 |
| *supardii* | sup | JN117554 – JN117561 |
| Sect. *Pardalopetalum* |  |  |
| *dianthum* | di | JN117355 – JN117362 |
| *haynaldianum* | hay | JN117411 – JN117418 |
| *lowii* | low | JN117451 – JN117458 |
| *parishii* | par | JN117490 – JN117497 |
| Sect. *Barbata* |  |  |
| *acmodontum* | acmo | JN117291 – JN117298 |
| *curtisii* | curt | JN117331 – JN117338 |
| *dayanum* | day | JN117339 – JN117346 |
| *hennisianum* | hen | JN117419 – JN117426 |
| *purpuratum* | purp | JN117506 – JN117513 |
| *sangii* | sang | JN117530 – JN117537 |
| *sukhakulii* | suk | JN117546 – JN117553 |
| *venustum* | ven | JN117570 – JN117577 |
| *wardii* | war | JN117586 – JN117593 |
