# Supplementary figures and images for: Dynamic distribution patterns of ribosomal DNA and chromosomal evolution in Paphiopedilum, a lady's slipper orchid
Source: BMC Plant Biol. 2011 Sep 12;11:126. doi: 10.1186/1471-2229-11-126 (PMC3184063; doi:10.1186/1471-2229-11-126)

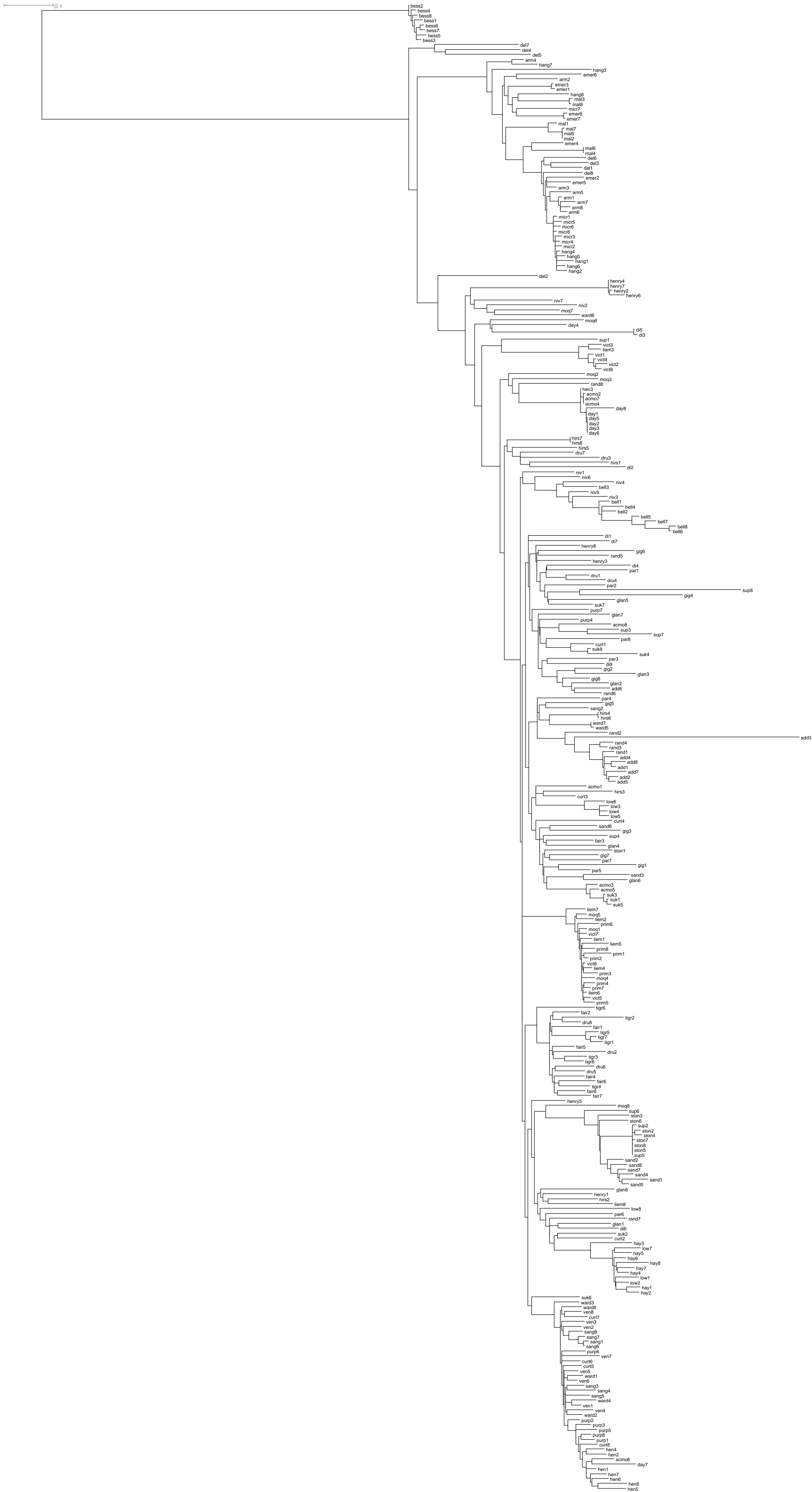

Supplement: Additional file 2 — 5S-NTS sequences: the single tree of maximum likelihood [file 1471-2229-11-126-S2.PDF]

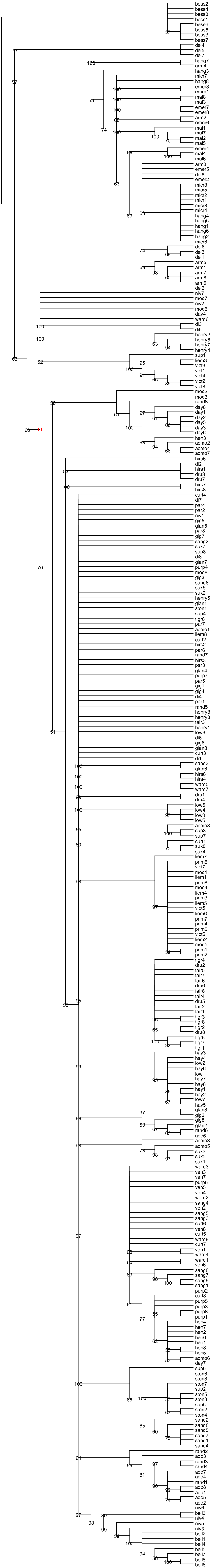

Supplement: Additional file 3 — 5S-NTS sequences: the majority-rule consensus tree based on 100 bootstrap replications [file 1471-2229-11-126-S3.PDF]
